# Acute effect of citrulline malate on flow-mediated dilation and serum pharmacodynamics in healthy young males

**DOI:** 10.3389/fphys.2026.1773582

**Published:** 2026-03-06

**Authors:** Johan Grannes, Nigel A. Callender, Adam M. Gonzalez, Jonny Hisdal, Fredrik T. Vårvik, Thomas Bjørnsen

**Affiliations:** 1 Department of Sports Science and Physical Education, University of Agder, Kristiansand, Norway; 2 Department of Vascular Surgery, Oslo University Hospital, Oslo, Norway; 3 Department of Allied Health and Kinesiology, Hofstra University, Hempstead, NY, United States; 4 Institute of Clinical Medicine, Faculty of Medicine, University of Oslo, Oslo, Norway; 5 Norwegian Olympic and Paralympic Committee and Confederation of Sports, Oslo, Norway; 6 Department of Education and Sports Science, University of Stavanger, Stavanger, Norway

**Keywords:** arginine, blood flow, citrulline, flow-mediated dilatation, nitric oxide

## Abstract

**Introduction:**

The use of ergogenic compounds has gained increasing popularity among individuals who wish to improve performance and recover faster from their workouts. Among these products is citrulline malate (CitMal), a popular dietary supplement that is suggested to enhance nitric oxide (NO)-mediated vasodilation and muscle blood flow.

**Methods:**

To evaluate effects on arterial function, flow-mediated dilation (FMD) of the brachial artery during active hyperemia was measured in 12 healthy, recreationally active males (23 ± 3 years) before and after (60- and 120-min post) consuming either 6 g CitMal, 12 g CitMal, or a taste-matched placebo. The study used a randomized, double-blind, placebo-controlled, within-subject counterbalanced crossover design with ≥7-day washouts.

**Results:**

Repeated measures ANOVA revealed no significant interaction (p = 0.315) or time effect (p = 0.649) in corrected FMD% at 60- and 120-min after intake of placebo, 6 g CitMal, and 12 g CitMal. There were also no significant differences (p = 0.301) between doses at any timepoint. A subgroup of six participants completed two additional visits to assess the effect of CitMal ingestion on serum markers involved in NO production. Over 120-min post-consumption, both doses significantly increased peak serum concentrations of citrulline (6 g: 504.7 ± 139.7; 12 g: 881.9 ± 216.7 μM), arginine (6 g: 70.2 ± 20.4; 12 g: 101.8 ± 36.2 μM), and ornithine (6 g: 27.9 ± 14.2; 12 g: 56.5 ± 30.0 μM) from baseline (all p < 0.001), with greater increases following 12 g (all p < 0.05). Likewise, arginine-to-dimethylarginine ratios (SDMA and ADMA) increased from baseline (SDMA, 6 g: 114.1 ± 24.2; 12 g: 166.2 ± 43.7; ADMA, 6 g: 119.2 ± 31.8; 12 g: 169.1 ± 29.1; all p < 0.001), with greater increases following 12 g (p < 0.05).

**Discussion:**

Collectively, these findings suggest that neither 6 g nor 12 g of CitMal significantly enhance FMD within 120 min, despite marked increases in biochemical markers favorable to NO production. To our knowledge, this is the first study to compare acute doses of CitMal up to 12 g in relation to brachial artery FMD. These results indicate that acute vascular responses to CitMal may be limited by physiological ceiling effects and that potential vascular benefits may depend on longer-term supplementation, the presence of an exercise stimulus, or populations with impaired endothelial function.

## Introduction

L-citrulline is a non-essential and non-proteogenic amino acid found primarily in watermelon that functions as a precursor of L-arginine (Gonzalez and Trexler, 2020). Via its role as an intermediate of the urea cycle, L-citrulline is converted to L-arginine for subsequent use by vascular endothelial cells to synthesize nitric oxide (NO) via the L-arginine-NO pathway (Gonzalez et al., 2023). NO promotes relaxation of vascular smooth muscle and vasodilation by activating the enzyme guanylate cyclase. This causes the production of cyclic guanosine monophosphate (cGMP) from guanosine triphosphate (GTP) which decreases sarcoplasmic calcium levels leading to smooth muscle relaxation and vasodilation (Gonzalez et al., 2023; Figueroa et al., 2020). Over the past decade, research on L-citrulline as a dietary supplement to enhance endothelial function and exercise performance has grown due to its potential NO-mediated effects on blood flow, although the findings have been mixed (Gonzalez and Trexler, 2020; Gonzalez et al., 2023). In these studies, L-citrulline is often delivered as citrulline malate (CitMal), a combination of L-citrulline and malate. Malate, an intermediate in the citric acid cycle, is thought to potentially enhance ATP production, though this has not been substantiated by experimental evidence (Gough et al., 2021).

L-citrulline supplementation has been shown to be more efficient than direct L-arginine supplementation for elevating endogenous L-arginine bioavailability and NO-dependent signalling (Schwedhelm et al., 2008; Kim et al., 2015). This is likely attributable to orally consumed L-arginine undergoing significant catabolism in the gut by the enzyme arginase, in addition to subsequent first-pass metabolism (Bahadoran et al., 2021). L-citrulline, on the other hand, is well-absorbed and converted to L-arginine to promote NO synthesis. Several human studies have reported an increase in plasma citrulline and arginine concentrations following the intake of L-citrulline, CitMal, and watermelon juice (Schwedhelm et al., 2008; Bailey et al., 2015; Collins et al., 2007; Mandel et al., 2005; Moinard et al., 2008; Rougé et al., 2007; Sureda et al., 2010; Suzuki et al., 2016). Additionally, Moinard et al. (Moinard et al., 2008) found that L-citrulline supplementation increased plasma citrulline and arginine levels in a dose dependent manner up to 10 g, with peak arginine concentrations occurring ∼90–100-min following ingestion.

Few studies have examined the effect of L-citrulline or CitMal supplementation on flow-mediated dilation (FMD) and arterial blood flow (Kim et al., 2015; Cutrufello et al., 2015; Rogers et al., 2020; Trexler et al., 2020). Cutrufello et al. (2015) did not observe any changes in FMD at 60- or 120-min following 6 g L-citrulline, while Rogers et al. (2020) reported significant increases in FMD 60-min after 8 g CitMal in healthy, physically active young adults. Trexler et al. (2020) also reported no effect on muscle blood flow before or during resistance exercise when an 8 g dose of CitMal was consumed 120-min prior. Consequently, the present study aimed to assess the acute impact of 6 g and 12 g CitMal supplementation on brachial artery FMD at 60- and 120-min after ingestion. Furthermore, in a subgroup of participants, the serum pharmacodynamics of CitMal were examined, including measurements of L-citrulline, L-arginine, and L-ornithine. Additionally, serum measurements included endogenous NO synthesis inhibitors–asymmetric dimethylarginine (ADMA) and symmetric dimethylarginine (SDMA) – which have been shown to decrease L-arginine bioavailability and its subsequent conversion to NO, potentially attenuating FMD (Tain and Hsu, 2017). It was hypothesized that CitMal would increase FMD and serum markers in a dose-dependent manner.

## Materials and methods

### Participants

Twelve healthy males (23 ± 3 years [mean ± SD], 181.8 ± 5.8 cm, 84.4 ± 13.1 kg, systolic blood pressure [BP]: 119.2 ± 8.1 mmHg, diastolic BP: 62.5 ± 4.3 mmHg) volunteered to participate in this study. *A priori* analysis (α = 0.05, β = 0.8), anticipating a large effect size (Cohen’s *f* = 0.46), based upon changes in FMD in previous work (Rogers et al., 2020) indicated that a minimum of 12 participants was necessary for the detection of statistical significance. All participants were recreationally active (participating in moderate-to-high intensity exercise 3 to 5 times per week) and had a normal BMI (18.5–24.9 kg/m^2^). All were free from known medical conditions, and none were using medications, tobacco products, or dietary supplements that would impact study endpoints. Following an explanation of all procedures, risks, and benefits, each participant provided his written informed consent before participation in this study. The present study was prospectively reviewed by the Regional Committees for Medical and Health Research Ethics in Norway (REK; application no. 358636). REK formally concluded that the project falls outside the scope of the Norwegian Health Research Act, as its primary aim was to investigate physiological mechanisms in healthy individuals, rather than to generate new knowledge about health or disease. As the study was explicitly assessed and exempted from classification as health research or a clinical trial by the national ethics authority, it was not registered in a clinical trial registry. However, the study was subsequently approved by the Faculty Research Ethics Committee at the University of Agder and the Norwegian Centre for Research Data (reference no. 376940), and conducted in accordance with institutional ethical guidelines and the Declaration of Helsinki.

### Research design

This study was conducted as a randomized, double-blind, placebo-controlled experiment using a within-subject counterbalanced crossover design. All participants reported to the laboratory on 3 separate occasions with a 7-day washout period between each visit. Participants completed all experimental conditions in a randomized, counterbalanced order, such that the sequence of placebo, 6 g, and 12 g citrulline malate administration was varied across participants to minimize order and carryover effects. Brachial artery FMD was assessed at baseline (prior to supplement intake), 60-min, and 120-min after supplementation with either 6 g CitMal, 12 g CitMal, or a placebo. All testing was conducted in the morning or early afternoon and at the same time of the day (±1 h) for each participant. Participants arrived at the laboratory for each experimental trial following a minimum 4-h fasted state, having abstained from caffeine for the past 12 h and avoiding strenuous exercise for the previous 24 h. Those participating in the early afternoon were instructed to consume their typical breakfast on the morning of each visit and to record and replicate this meal across all trials. However, dietary nitrate or antioxidant intake was not specifically assessed or restricted. Additionally, participants were requested to maintain normal dietary, sleep, and exercise patterns while enrolled in the study.

A subgroup of 8 participants (24 ± 2 years, 180.9 ± 3.3 cm, 82.5 ± 7.1 kg) undertook two additional visits for the analysis of serum pharmacodynamics following CitMal ingestion. In this serum pharmacodynamic sub-analysis, participants only completed two additional visits involving 6 g and 12 g citrulline malate. A placebo trial was not included in the sub-analysis to reduce participant burden. During the trials, whole blood samples were collected at baseline, 30-, 60-, 90-, and 120-min after supplementation with either 6 g or 12 g CitMal and analyzed for serum concentration of L-citrulline, L-arginine, L-ornithine, SDMA, and ADMA. Like FMD measures, these trials were conducted at the same time of the day (±1 h) with a 7-day washout period between each visit. Participants were provided with the same dietary and exercise guidelines as prior to the FMD sessions.

### Supplementation

Supplements were consumed as a 500 mL beverage containing either 6 g CitMal, 12 g CitMal, or a placebo. A commercially available CitMal supplement with a purported 2:1 ratio of L-citrulline and malate (Trade Ingredients, South Shields, United Kingdom) was used in the present study. Previously, the citrulline:malate ratio for this product has been reported at 1.92:1 ± 0.10 via nuclear magnetic resonance spectroscopy (Chappell et al., 2018). Placebo contained 1.5 g of citric acid to match the taste of the CitMal supplement. Both drinks were colorless and visually indistinguishable. Blinded pilot tests were conducted, and subjects could not differentiate between CitMal and placebo beverages. Randomization and supplement preparation was performed by a third-party researcher who did not take part in the data collection or analysis of the data. Codes were revealed only after all data processing and statistical analyses were finalized, ensuring that all data processing were conducted by investigators blinded to treatment condition.

### Brachial artery flow-mediated dilation

FMD measurements were assessed by the same operator (between-day intraclass correlation coefficient [ICC] for baseline diameter: 0.76) in accordance with evidence-based recommendations presented by Thijssen et al. (2019). Upon each assessment, participants were positioned in a supine position with the right arm placed in a supported, ∼80-degree position of abduction. The brachial artery was insonated 5–10 cm proximal to the elbow crease using a 9 MHz linear probe (9L Probe & Vivid E95; Vingmed GE, Horten, Norway), and once the image was optimized for the best possible delineation of the near and far vessel walls, the probe was fixed in position using a custom probe holder.

Following a period of quiet rest, a 120-s baseline B-mode ultrasound recording of the artery was obtained. Subsequently, a forearm occlusion cuff (VascuLab; Stranden Instruments, Ålesund, Norway) was rapidly inflated to 250 mmHg in a position 2 cm distal to the elbow crease. Arterial occlusion was maintained for 5-min before rapidly deflating. Thirty seconds prior to cuff deflation, ultrasound recording was re-commenced and continued for 3-min post-deflation. Similar probe placement between each trial was ensured using skin surface markings and identification of on-screen anatomical landmarks.

### Analysis of flow-mediated dilation

Recordings of each FMD sequence were exported in DICOM file format and analyzed using semi-automated edge detection software (Brachial Analyzer; Medical Imaging Applications, Iowa, United States) sampled at 10 Hz. All images were manually checked by the same observer to ensure correct detection of the near and far arterial walls during each analysis sequence. D_base_ and D_peak_ (mm) were established from the edge-detection software where D_base_ is the mean baseline arterial diameter during the 120-s interval preceding cuff inflation and D_peak_ is the maximal diameter attained during the 3-min post arterial occlusion phase. Previous work has established a negative correlation between D_base_ and FMD response that can lead to an unintentional bias in the interpretation of the data (Atkinson, 2014). In accordance with recommendations by Atkinson (Atkinson, 2014), the natural logarithm was therefore calculated for D_base_ and D_peak_ and a predictive regression analysis was conducted to find an appropriate exponent for the relationship between these two variables. Thereafter, corrected FMD% was calculated with the equation: [(lnD_peak_ - lnD_base_)/lnD_base_^0.96^)] × 100 (Atkinson, 2014).

### Blood sampling and analysis

Blood samples were obtained from a superficial forearm vein using a 21G Vacutainer system and collected in 5 mL serum-separating tubes (BD Vacutainer SST; Plymouth, United Kingdom) using standard procedures. Blood samples were gently agitated as per manufacturer guidance, allowed to clot for 15-min, and subsequently centrifuged (Labofuge 400R, Thermo Electron Corporation) at 1500 rpm for 10-min. Thereafter, samples were checked for inadequate separation, clots, or other abnormalities and transferred by pipette for prompt storage at −80 °C.

Serum levels of citrulline, arginine, ornithine, SDMA, and ADMA were later analyzed by a third-party laboratory (Bevital AS, Bergen, Norway) using a multianalyte quantification method based on high-performance liquid chromatography tandem mass spectrometry (HPLC-MS/MS) as described by Midttun et al. (2013). Limits of detection (LOD) were below typical serum concentrations in healthy individuals for citrulline (LOD: 4 μmol/L; intraclass correlation coefficient [ICC]: 0.62), ornithine (LOD: 0.5 μmol/L; ICC: 0.55), arginine (LOD: 0.25 μmol/L; ICC: 0.53), ADMA (LOD: 0.08 μmol/L; ICC: 0.53), and SDMA (LOD: 0.08 μmol/L; ICC: 0.62).

The ratios between arginine and its methylated derivatives SDMA and ADMA were calculated to examine if any increase in arginine levels would yield a disproportional increase in these biomarkers. Both SDMA and ADMA have been shown to negatively impact NO production by decreasing the intracellular bioavailability of arginine (Bode-Böger et al., 2006) and inhibit endothelial NO synthase (eNOS) activity (Leone et al., 1992). Thus, a decrease in these ratios could indicate reduced eNOS-activity and subsequent NO production. Additionally, as arginine converts to both NO and ornithine (Bahadoran et al., 2021), the ratios between arginine and ornithine were calculated. Collectively, changes in these ratios could provide a better understanding of the influence of CitMal on NO production.

### Statistical analyses

Normality of distribution was assessed using Shapiro-Wilk tests, with non-normally distributed variables logarithmically transformed prior to analysis. Levene’s and Mauchly’s tests were applied to assess the assumptions of homoskedasticity and sphericity respectively, and the Greenhouse-Geisser correction applied when required. Missing data were excluded in a listwise manner. Two data points were missing in the serum biomarker subgroup and were handled using listwise deletion. Given the minimal extent of missingness and its random distribution, this approach was considered appropriate for preserving analytic simplicity without introducing bias. Two-way (dose [placebo, 6 g CitMal, 12 g CitMal] × time [baseline, 60-min, and 120-min]) repeated measures analysis of variance (ANOVA) were performed on corrected FMD%. For the subgroup, two-way (dose [6 g CitMal, 12 g CitMal] × time [baseline, 30-, 60-, 90-, and 120-min]) repeated measures ANOVA were performed on serum amino acids and their ratios. Following any significant F-ratio, *post-hoc* comparisons were made using Bonferroni adjusted estimated marginal means. Significance was accepted at an alpha level of *p* < 0.05. All data are presented as mean ± standard deviation with ANOVA effect sizes estimated using the partial eta-squared method (ηp^2^), interpreted as small = 0.01, medium = 0.06, and large = 0.14, and pairwise effect sizes estimated using Cohen’s *d*, interpreted as small = 0.2, medium = 0.5, and large = 0.8 (Lakens, 2013). All statistical analyses were performed using IBM SPSS version 30.0 (IBM Corp., Armonk, NY, United States).

## Results

All participants (n = 12) completed all FMD measurement trials. Eight participants completed blood measurements, with two participants excluded from analyses due to issues with the blood draw. As a result, blood analysis was conducted on data from six participants.

### Flow-mediated dilation

**FIGURE 1:**
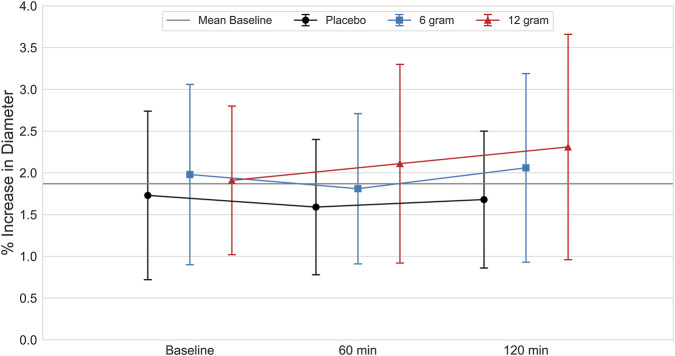
Percentage increase in diameter of the brachial artery (FMD%) after deflation of an occluding cuff, measured at baseline, 60-min and 120-min after ingestion of placebo, 6 g and 12 g of CitMal. Values are expressed as mean ± SD.

No significant main effect of time (p = 0.649; ηp^2^ = 0.013) or dose (p = 0.301; ηp^2^ = 0.070) was apparent for corrected FMD% (Figure 1). Similarly, no significant interaction was present between the timepoint of corrected FMD% assessment and dose (p = 0.315; ηp^2^ = 0.019). As a prespecified analysis to better integrate baseline vascular characteristics with the primary outcomes, regression analysis revealed a significant negative correlation (r = −0.60, *R* ^2^ = 0.36, p < 0.001) between baseline diameter of the brachial artery (4.66 ± 0.61 mm) and corrected FMD%, with participants with larger artery diameter (>5 mm) showing a lower relative dilatory response (2%–3%).

### Serum concentrations

**FIGURE 2:**
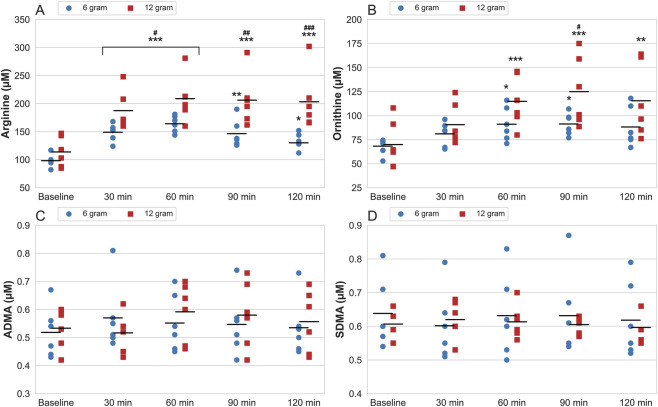
Serum levels of arginine **(A)**, ornithine **(B)**, asymmetric dimethylarginine (ADMA) **(C)** and symmetric dimethylarginine (SDMA) **(D)** measured at baseline and 30–120-min after consumption of 6 and 12 g CitMal. Values are presented as data plots with mean (black horizontal line) for each measurement time. * = statistically significantly different from baseline (* = p < 0.05, ** = p < 0.01, *** = p < 0.001). # = significantly different from 6 g at the same measurement time (# = p < 0.05, ## = p < 0.01, ### = p < 0.001).

**FIGURE 3:**
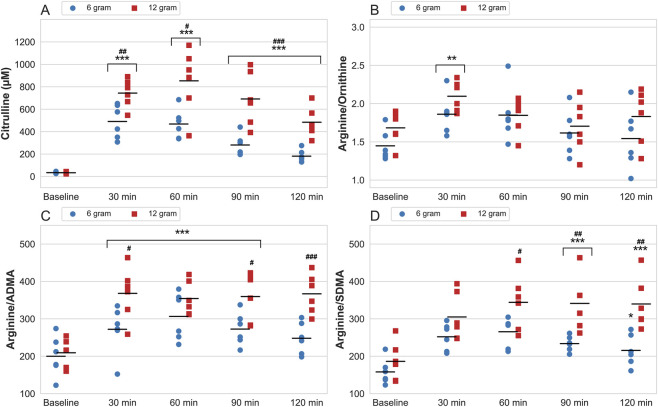
Serum levels of citrulline **(A)** and the ratios between arginine and ornithine **(B)**, asymmetric dimethylarginine (ADMA) **(C)** and symmetric dimethylarginine (SDMA) **(D)**, measured at baseline and 30–120-min after ingestion of 6 and 12 g CitMal. Values are expressed as data plots with mean (black horizontal line) for each measurement time and each dose. * = statistically significantly different from baseline (* = p < 0.05, ** = p < 0.01, *** = p < 0.001). # = significantly different from 6 g at the same measurement time (# = p < 0.05, ## = p < 0.01, ### = p < 0.001).

Serum concentrations of citrulline, arginine, ornithine, ADMA, and SDMA are presented in Figures 2, 3. Analysis of variance for serum citrulline concentration indicated a significant main effect of time (p < 0.001, ηp^2^ = 0.966) and dose (p < 0.001, ηp^2^ = 0.742), accompanied by a significant time × dose interaction (p < 0.001, ηp^2^ = 0.456). Peak serum concentration for the 6 g dose occurred 30-min post-ingestion (change from baseline at peak: 469.8 ± 143.5 μM, p < 0.001) and at 60-min following the 12 g dose (change from baseline at peak: 810.9 ± 264.5 μM, p < 0.001).

Significant main effects for time (p < 0.001, ηp^2^ = 0.851) and dose (p = 0.007, ηp^2^ = 0.467) were present for serum arginine accompanied by a significant time × dose interaction (p = 0.018, ηp^2^ = 0.275). Arginine showed a greater increase after ingestion of 12 g compared to 6 g CitMal at all measurement times, peaking 60-min after intake for both doses (change from baseline at peak 6 g: 70.0 ± 21.3 μM, p < 0.001; 12 g: 96.9 ± 34.9 μM, p < 0.001).

Serum ornithine concentration showed a significant main effect of time (p < 0.001, ηp^2^ = 0.712), but not dose (p = 0.145, ηp^2^ = 0.168), with no significant interaction present (p = 0.143, ηp^2^ = 0.150). Peak concentration occurred 60-min following ingestion of the 6 g dose (change from baseline at peak: 24.2 ± 12.8 μM, p = 0.035) and at 90-min following the 12 g dose (change from baseline at peak: 51.6 ± 30.7 μM, p < 0.001).

Serum ADMA concentration showed a significant main effect for time (p = 0.015, ηp^2^ = 0.223), but not dose (p = 0.613, ηp^2^ = 0.022), and was accompanied by a significant time × dose interaction (p = 0.022, ηp^2^ = 0.223). Upon *post-hoc* testing, no statistically significant differences were present.

No significant differences were observed in serum levels of SDMA for time (p = 0.635, ηp^2^ = 0.051) or dose (p = 0.826, ηp^2^ = 0.004), and no significant time × dose interaction was present (p = 0.959, ηp^2^ = 0.013).

### Ratio values

The ratio of arginine/ornithine, arginine/ADMA, and arginine/SDMA are presented in Figure 3. The ratio between arginine/ornithine showed a significant main effect for time (p = 0.002, ηp^2^ = 0.405), but not dose (p = 0.444, ηp^2^ = 0.050), with no significant time × dose interaction present (p = 0.217, ηp^2^ = 0.111). Serum arginine/ornithine ratio increased 30-min after ingestion of both 6 and 12 g CitMal (change from baseline at peak 6 g: 0.4 ± 0.2, p = 0.005; 12 g: 0.4 ± 0.3, p = 0.005).

Serum arginine/ADMA ratio showed significant main effect of time (p < 0.001, ηp^2^ = 0.834) but not dose (p = 0.083, ηp^2^ = 0.229). A significant time × dose interaction was present (p = 0.001, ηp^2^ = 0.313). Peak arginine/ADMA ratio occurred at 60-min after ingestion of 6 g CitMal (change from baseline at peak: 119.7 ± 46.7, p < 0.001) and 30-min after ingestion of 12 g CitMal (change from baseline at peak: 152.2 ± 39.9, p < 0.001).

Serum arginine/SDMA ratio showed a significant main effect for time (p < 0.001, ηp^2^ = 0.831) and dose (p = 0.001, ηp^2^ = 0.426), and was accompanied by a significant time × dose interaction (p = 0.007, ηp^2^ = 0.250). Peak arginine/SDMA occurred at 60-min after ingestion of both 6 g and 12 g CitMal (change from baseline at peak 6 g: 105.0 ± 26.5, p < 0.001; change from baseline at peak 12 g: 151.9 ± 47.4, p < 0.001).

## Discussion

The primary objective of this study was to assess the acute, dose-dependent effects of CitMal supplementation on brachial artery FMD, an endothelial-dependent measure of arterial responsiveness to shear stress. No significant changes in corrected FMD% were observed at either 60- or 120-min following ingestion of 6 g or 12 g CitMal in healthy young adults at rest. These findings align with those of Cutrufello et al. (2015), who also reported no significant changes in FMD at similar time points after ingestion of 6 g L-citrulline. In contrast, Rogers et al. (2020) observed a significant 25% increase in FMD from baseline 60 min after ingestion of 8 g CitMal, compared to a 0.6% increase with placebo. The reason for these conflicting results remains unclear. However, while all studies involved a comparable sample of recreationally active young adults, Rogers et al. (2020) did supplement with a slightly higher dose of L-citrulline than Cutrufello et al. (2015). To the authors’ knowledge, the present investigation is the first to examine the dose-response relationship of CitMal supplementation (6 g vs. 12 g) on FMD, as well as the first to evaluate a dose exceeding 8 g. The relative increase in corrected FMD% seemed to favor 12 g CitMal at 120 min after ingestion, with an average increase above baseline of 21%, compared to 4% with 6 g and −2% with placebo at the same time point. Importantly, this trend did not reach statistical significance and should therefore be interpreted cautiously. It may suggest a potential time- and dose-dependent vascular response, warranting further investigation in future studies with greater statistical power.

Regression analyses from this study revealed a significant negative correlation between baseline diameter of the brachial artery and FMD. This decrease in FMD has also been demonstrated in previous research (Birk et al., 2012; Tinken et al., 2008; Tinken et al., 2010) and may suggest remodeling of muscular arteries with increasing training experience to accommodate for larger blood flow requirements. In line with this, we could speculate a simultaneous decrease in the production and/or response to NO, and thus a reduced response to NO-boosting supplements such as CitMal among trained participants. A single dose of CitMal supplementation may also have limited impact on blood flow in healthy young active adults due to the natural constraints of vascular elasticity and the efficiency of normal endothelial regulation (Allerton et al., 2018).

Similar to our findings, Trexler et al. (2019) reported no significant changes in indices of femoral artery blood flow 120 min following ingestion of 8 g CitMal in young healthy active individuals. Furthermore, a recent meta-analysis of six studies reported no acute postprandial effects of L-citrulline supplementation on FMD and vascular function markers in healthy individuals (Smeets et al., 2022). Nevertheless, the meta-analysis found that longer-term L-citrulline supplementation (ranging from 1 week to 4 months) led to a 0.9% improvement in FMD (95% confidence interval: 0.7–1.1, p < 0.001), suggesting potential vascular benefits that could positively influence cardiovascular health outcomes, especially in clinical populations such as those with obesity, heart failure, coronary artery disease or type 2 diabetes. Additionally, Maharaj et al. (2022) reported that 4 weeks of L-citrulline supplementation (10 g) increased FMD compared to a placebo (Δ 1.4% ± 2.0% vs. Δ −0.5% ± 1.7%) in hypertensive postmenopausal women.

The subgroup analysis of serum pharmacodynamics revealed a dose-dependent increase in serum citrulline, with the 12 g CitMal dose resulting in higher concentrations throughout the 120-min postprandial period compared to the 6 g dose. This is consistent with previous studies indicating that intestinal absorption of L-citrulline does not seem to be a limiting step, even at relatively high doses (Schwedhelm et al., 2008; Moinard et al., 2008). Moinard et al. (2008) reported a 10- to 100-fold increase in plasma citrulline concentration after ingestion of 2–15 g L-citrulline. Consistent with previous studies investigating the dose-response effects of L-citrulline on plasma arginine (Schwedhelm et al., 2008; Moinard et al., 2008), the current study also demonstrated a dose-dependent increase in serum arginine following supplementation, despite differences in absolute values. The highest serum concentration of arginine (207 ± 37 μM) was measured 60-min after ingestion of 12 g CitMal, which is an 88% increase from baseline. This further supports the growing body of evidence (Schwedhelm et al., 2008; Moinard et al., 2008) that L-citrulline is an effective precursor to arginine, with its effects occurring in a dose-dependent manner.

Although arginine serves as the primary substrate for NO production through the L-arginine-NO pathway, it is also metabolized by arginase into ornithine as part of the urea cycle (Morris, 2016). Therefore, the arginine-to-ornithine ratio serves as an important marker for evaluating its potential role in NO production. In the current study, both 6 and 12 g CitMal significantly elevated serum levels of ornithine compared to baseline values from 60- to 120-min postprandial. Despite this increase in ornithine, the arginine-to-ornithine ratio was elevated at all time points, with a significant peak at 30 min postprandial (1.9 ± 0.2 compared to 1.6 ± 0.2 at baseline), suggesting a greater allocation of arginine toward NO synthesis rather than the urea cycle (Morris, 2016). Although our study assessed serum arginine and ornithine levels, previous research (Moinard et al., 2008) using plasma measurements shows results that align closely with ours, despite differences in absolute values.

To further investigate any potential interference on NO synthesis, the methylated arginine derivatives, ADMA and SDMA, were measured and their ratio to arginine was calculated. Both SDMA and ADMA have been shown to negatively impact NO production by decreasing the intracellular bioavailability of arginine (Bode-Böger et al., 2006) and inhibit eNOS activity (Leone et al., 1992). Thus, a decrease in the arginine-to-ADMA and arginine-to-SDMA ratios could indicate reduced eNOS-activity and subsequent NO production.

Despite significant increases in arginine concentrations, ADMA and SDMA remained relatively unchanged after both 6 g and 12 g of CitMal. Previous studies using supplemental arginine have similarly reported no changes in serum or plasma levels of ADMA and SDMA, despite significant increases in arginine concentrations (Alvares et al., 2012; Zarezadeh et al., 2020). The arginine-to-ADMA and arginine-to-SDMA ratios increased following ingestion of both 6 and 12 g CitMal, suggesting that arginine concentration and bioavailability would theoretically provide eNOS with substantially more substrate for NO production (Tain and Hsu, 2017). This supports the hypothesis that the absence of an FMD response in our study may reflect limitations in translating increased substrate availability into functional vasodilation.

Several limitations should be considered when interpreting the findings. First, these findings are limited to healthy, recreationally active young adult males and may not be generalizable to other populations such as older adults, individuals with cardiovascular risk factors and impaired endothelial function, or women. Future studies should explore whether similar responses occur in more diverse cohorts. Second, although our sample size was based on prior data showing a large acute effect of citrulline on FMD (Rogers et al., 2020), we acknowledge that such effect sizes may be optimistic, particularly in healthy populations with preserved endothelial function. Thus, the study may have been underpowered to detect more modest but physiologically relevant effects. Third, it is important to note that the serum pharmacodynamic sub-study did not include a placebo control condition. While this design choice was made to reduce participant burden and venous sampling frequency, it limits the ability to isolate supplementation effects from time-dependent changes due to circadian rhythms, fasting, or postural influences. As such, findings from this exploratory sub-analysis should be interpreted with appropriate caution. Finally, given the lack of dietary standardization or nitrate control, variations in habitual dietary intake may have influenced endothelial function or NO-related biomarkers and should be considered in future studies.

## Conclusion

This study offers valuable insights into the acute effects of CitMal on vascular function and nitric oxide precursors by assessing endothelially mediated arterial vasodilation and serum biomarker responses following acute ingestion of 6 and 12 g of CitMal in healthy young males. Results show that although both doses caused a significant increase in serum arginine levels, they did not produce a meaningful improvement in FMD within 2 h post-ingestion. These findings suggest that despite increased arginine availability, acute CitMal consumption does not enhance endothelial-dependent arterial vasodilation at rest. Nevertheless, these null findings should be interpreted with caution, as the study may have lacked statistical power to detect smaller, yet meaningful, changes in endothelial function. In addition, our conclusions apply specifically to young, healthy males with preserved endothelial function and may not extend to clinical or aging populations. To better understand CitMal’s potential impact on vascular function, future research should investigate its long-term effects and efficacy under both resting and active conditions across diverse populations, while maintaining strict dietary control. Such studies will help determine whether CitMal has practical applications for vascular health and athletic performance.

## Data Availability

The raw data supporting the conclusions of this article will be made available by the authors, without undue reservation.
